# Effects of Tool Novelty and Action Demands on Gaze Searching During Tool Observation

**DOI:** 10.3389/fpsyg.2020.587270

**Published:** 2020-11-23

**Authors:** Yoshinori Tamaki, Satoshi Nobusako, Yusaku Takamura, Yu Miyawaki, Moe Terada, Shu Morioka

**Affiliations:** ^1^Department of Rehabilitation, Kohnan Hospital, Shiga, Japan; ^2^Graduate School of Health Sciences, Kio University, Nara, Japan; ^3^Neurorehabilitation Research Center, Kio University, Nara, Japan; ^4^Department of Rehabilitation for the Movement Functions, Research Institute, National Rehabilitation Center for Persons With Disabilities, Saitama, Japan; ^5^Research Fellow of Japan Society for the Promotion of Science, Tokyo, Japan; ^6^Department of Rehabilitation Medicine, Keio University School of Medicine, Tokyo, Japan; ^7^Department of Rehabilitation, Murata Hospital, Osaka, Japan

**Keywords:** technical reasoning, tool use, tool novelty, action demands, gaze

## Abstract

Technical reasoning refers to making inferences about how to use tools. The degree of technical reasoning is indicated by the bias of the gaze (fixation) on the functional part of the tool when in use. Few studies have examined whether technical reasoning differs between familiar and unfamiliar novel tools. In addition, what effect the intention to use the tool has on technical reasoning has not been determined. This study examined gaze shifts in relation to familiar or unfamiliar tools, under three conditions (free viewing, lift, and use), among 14 healthy adults (mean age ± standard deviation, 29.4 ± 3.9 years). The cumulative fixation time on the functional part of the tool served as a quantitative indicator of the degree of technical reasoning. The two-way analysis of variance for tools (familiar and unfamiliar) and conditions (free viewing, lift, and use) revealed that the cumulative fixation time significantly increased under free viewing and use conditions, compared to lift conditions. Relative to the free viewing condition, cumulative fixation time for unfamiliar tools significantly decreased in the lift condition and significantly increased in the use condition. Importantly, the results showed that technical reasoning was performed in both the use and the free viewing conditions. However, technical reasoning in the free viewing condition was not as strong as in the use condition. The difference between technical reasoning in free viewing and use conditions may indicate the difference between automatic and intentional technical reasoning.

## Introduction

Humans live in environments surrounded by a variety of tools. In both daily (e.g., eating, cooking, and grooming) and occupational activities, tools are selected according to the intended use or the activity to be performed. Two reasoning systems are required when using such tools; the first system is semantic reasoning, which concerns itself with what to do, based on the functional knowledge of the tool (Osiurak, 2014); the second is technical reasoning, which is the ability to solve physical problems, especially regarding tool use, based on abstract physical principles (i.e., mechanical knowledge) acquired through experience (Osiurak, 2014; Osiurak et al., 2020). For example, semantic reasoning is performed when recalling the act of combing one’s hair while looking at a comb or searching for a comb – with the intention to use it. Technical reasoning is used when one alters their grip and movements to accommodate the size and shape of different combs. Technical reasoning ability is important when dealing with a novel tool, which has an unknown function or when employing a tool for something beyond its standard use (e.g., stirring coffee with a butter knife) (Osiurak et al., 2009; Osiurak, 2014).

Apraxia is a neurological condition that develops after a left hemisphere stroke, making it difficult to use tools. Apraxia symptoms vary widely and include gestures and pantomime disorders (Signoret and North, 1984). Notably, tool use challenges are often a problem during rehabilitation in living situations. Patients with tool use disorders have been found to struggle to guess the function of unfamiliar novel tools (Goldenberg and Hagmann, 1998) as well as the applied use of familiar daily tools (Osiurak et al., 2009, 2013). Moreover, technical reasoning involves the left inferior parietal lobe (IPL), an area that is often damaged in patients with apraxia (Osiurak et al., 2020). Thus, technical reasoning is an important ability for tool use.

Neuroimaging research shows that when a tool is observed by someone with no intention to use it, activity is seen in the brain areas responsible for tool recognition and in motor-related areas, like the premotor cortex (Lewis, 2006). This is physiological evidence that the visual representation of the tool automatically initiates the process that prepares for its potential use. Moreover, when observing with no intention to use, the observer’s spatial attention was attracted to the functional part: an act known as or the characteristic gaze (Roberts and Humphreys, 2011; Myachykov et al., 2013; Van Der Linden et al., 2015). This is considered the effect of affordances, which owes to characteristics like having functional knowledge of tools (Van Der Linden et al., 2015). Affordance refers to the potential behaviors afforded to a subject by the environment (Gibson, 1985). A study on gaze response showed that, in familiar daily tools with a functional and grasping part separated along the long axis, the first fixation was biased toward the center and the next was biased toward the functional part; this fixation bias on the functional part was deemed a higher-order affordance effect, based on functional knowledge of tools (Van Der Linden et al., 2015). Other reports on the free observation of the combination of familiar tools and objects showed that, when the combination was consistent, participants gazed more the grasping part of the tool, whereas when the combination was inconsistent, participants gazed more its functional part (Federico and Brandimonte, 2019, 2020). However, the characteristics of spatial attention or gaze response when observing an unfamiliar novel tool without the intention to use it remain unclear.

In an action situation, gaze data, especially fixation time, are used to quantify the degree of preparation for an object, like a tool, to be manipulated. This is because gaze control serves to collect information concerning actions when one uses objects and tools in everyday situations (Hayhoe et al., 2003; Land and Tatler, 2009). In a study that determined which part of the operation target one gazes at, the first fixation was the object’s center of gravity, and the second was the point where the object is gripped so the action may take place (Brouwer et al., 2009; Belardinelli et al., 2015). Notably, there are studies that presented familiar daily tools and unfamiliar novel tools with a functional and grasping part (separated along the long axis) that required a mime to lift and use the tool; in these cases, the gaze was biased toward the functional part of the tool when the use was requested. This effect has also been shown to be stronger with unfamiliar novel tools than with familiar daily tools (Belardinelli et al., 2016). The subject’s biased fixation on the functional part of the novel tool when requesting use indicates that they are actively trying to process the mechanical properties to guess how to use the tool. In other words, when we intend to use familiar everyday tools, we can easily understand what to do by looking at the functional parts. With unfamiliar novel tools, however, it is often necessary to observe functional parts and guess how to use them based on their shapes. This suggests that the degree of technical reasoning can be expressed through the duration of fixation on the functional part of the tool. However, they compared the intention to lift, in which technical reasoning was not required, and the intention to use, in which technical reasoning was required (Belardinelli et al., 2016). Yet, one may still be preparing for potential use when one observes tools without that explicit intention. Indeed, during this time, we may be opting to use the tool through automatic reasoning. Consequently, it is hypothesized that, with or without familiarity, gaze bias toward the functional part of the tool will take place. This is expected to be the case during free observation as well as during intention to use, more so than during intention to lift.

The study aims to determine the impact that the presence or absence of action intention has on technical reasoning in healthy subjects. To this end, we compared the cumulative fixation time to the functional part of the tool. This was achieved by observing familiar daily tools and unfamiliar novel tools under three conditions: free observation, demand for use, and demand for lift. The cumulative fixation time on the functional part of the tool served as a quantitative indicator of the degree of technical reasoning. Notably, previous studies have used cumulative fixation time as the result, and changes in gaze shift over time were not clear. To examine these differences in more detail, we confirmed the temporal gaze shift in addition to the cumulative fixation time. It is important to examine the impact of tool novelty and action demands on technical reasoning to understand the pathogenesis of apraxia and develop rehabilitation techniques.

## Materials and Methods

### Participants

The experiment involved 14 healthy adults (age: 29.4 ± 3.9 years old; 10 females and 4 males). Participants were right-handed and had normal vision with normal or corrective eyeglasses. This study was conducted with the approval of the Research Ethics Committee of Kio University. Based on the Declaration of Helsinki, we gave due consideration to subjects’ safety, fully explained the research methods and potential risks, and conducted the study with consent.

### Setting

In the experimental room, each subject took a seated position approximately 60 cm from the monitor presenting the stimulus image. To record eye movements, an eye tracker with a sampling frequency of 60 Hz (Tobii Pro X2-60: Toby Technology Co., Ltd., Tokyo) was installed at the bottom of the monitor. The monitor used a 17.3-inch laptop PC (HP ProBook 470 G2/CT Notebook PC: HP Japan, Tokyo) with a resolution of 1,920 × 1,080 pixels.

### Stimulus

Stimuli comprised 12 tool images: six general, everyday life tools (familiar tools) and six novel tools (unfamiliar tools). Each tool was lengthy, held horizontally, could be used with one hand, and had its functional and grip parts clearly separated along its axis. Each image was created using the image editing software Photoshop CC (Adobe Systems Inc., Tokyo), so they all had the same length. The center of the tool was set at a viewing angle of 5.15° above the center of the screen, displayed horizontally at 13.69°, and the grip was always on the left.

### Conditions

The tool images were viewed under three conditions. The free viewing conditions came first and only required participants to gaze at the screen. The lift conditions came second and required a pantomime of lifting the tool with the left hand. Finally came the third condition, which required a pantomime of using the tool with one’s left hand. Under both mime conditions, the examiner instructed the subject to perform the mime according to instructions, after the latter reached out to the monitor, assuming there was a tool in front of it. Under the use condition, the examiner requested that the subject use their imagination if they did not know how to use the tool.

### Procedures

**FIGURE 1:**
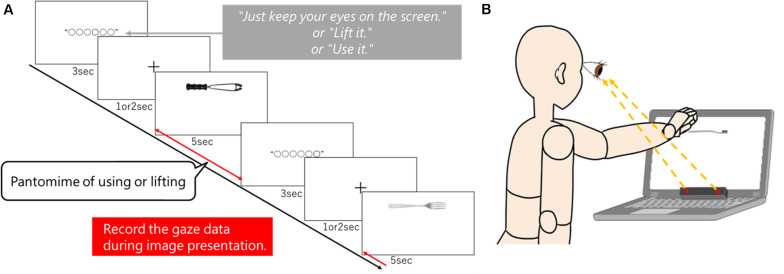
**(A)** Experimental protocol. The instructions for each condition were displayed for 3 s during each trial. Following this, a cross gaze point was randomly displayed in the center of the screen for 1 or 2 s, and then the tool image was displayed for 5 s. The tool images were displayed in the upper center of the screen, with the grasping part on the left and the functional part on the right. During each condition, 12 tool images were randomly displayed one time each. Under the use and lift conditions, subjects were instructed to perform an appropriate pantomime in front of the tool image display. We recorded participants’ eye movement recorded during the exercise. **(B)** Experimental scene. Participants took a seated position. In the lift and use conditions, participants reached for the screen and pantomimed the action of grasping the tool.

The experiment started with a nine-point calibration for each subject. Figure 1A shows the experimental procedure. In each set, the instruction associated with the condition (“Just keep your eyes on the screen”/“Lift it”/“Use it”) was displayed at the center of the screen for 3 s. Then, a cross point was displayed at the center of the screen for 1 or 2 s (random). After that, the tool image was presented for 5 s (see Figure 1A). The experiment was blocked for each condition. Furthermore, each condition had 12 tools displayed once in random order. Each condition was performed once, in succession, with a 1-min break after free viewing, lift, and use. Subjects underwent 36 trials each: three conditions using 12 tool images. Eye movements during the tool display were recorded, and results were recorded for each condition and tool type (familiarity). Each subject practiced each condition with another three tool images before performing this experiment. Tobii Studio (Tobii Technology Co., Ltd., Tokyo, Japan) was used to create the task and to measure eye movements. Figure 1B shows the actual experimental scene.

### Post-experiment Evaluation

(1) After the experiment, subjects drew rectangles of the range of the functional and of the grasping part of each tool. This amounted to a total of 12 for each of the tool images (Belardinelli et al., 2016). The image editing software Paint was used for the drawing.

(2) Subjects were asked to indicate their degree of familiarity with each tool on a scale of 1 to 5. Echoing previous studies (Belardinelli et al., 2016), each scale was set as follows, 5: I see it every week or every day, 4: I have seen it in the last month to a year, 3: I have seen it once or twice before, 2: I know it exists, and 1: I have never used it, seen it, or heard about it.

### Data Analysis

#### Visualization of Fixations

The minimum analysis time for eye movements was 1/60 of second; the target time was 5,000 ms, from the beginning to the end of each tool image presentation. Using the analysis software, Tobii Studio, heat maps were created, based on the fixation points of each tool. Using the Tobii Studio Clear View fixation filter, we set the minimum time required for a fixation at 100 ms. The velocity threshold selected was 100 pixels/16.6 ms, corresponding to a viewing angle of approximately 2° (Tobii Studio User’s Manual Version 3.4.5, 2020).

#### Region of Interest Settings and Cumulative Fixation Time on Functional Parts

**FIGURE 2:**
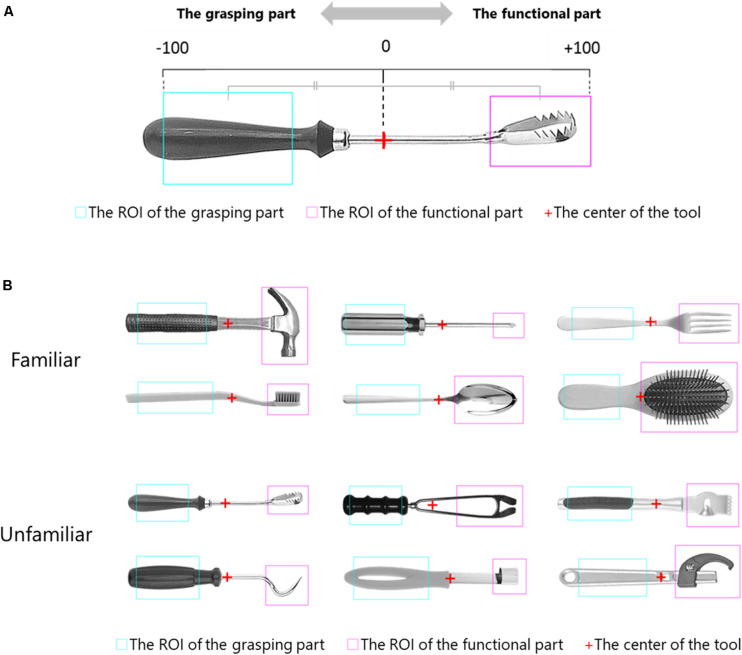
**(A)** An example of the region of interest (ROI) for the functional part, the ROI of the grasping part, and the standardization of horizontal coordinates from the tool center. The center of the tool was set at the midpoint (red +) of the center of the ROI for the functional part (the range surrounded by magenta lines) and the ROI for the grasping part (the range surrounded by blue lines), defined by the average of each subject. The horizontal coordinates of each line of sight were standardized from the center to the outer edge of each ROI, with the functional part as + 100 and the grasping part as −100. **(B)** All tool images, with the ROI for the grasping part, the ROI for the functional part, and the center of the visible tool. Top two rows: familiar tools; bottom two rows: unfamiliar tools. The ROI for the grasping part is indicated by the blue line, and the ROI of the functional part is indicated by the magenta line. The center of the tool is indicated by the red cross.

Following a previous study (Belardinelli et al., 2016), the region of interest (ROI) for each tool’s functional and grasp parts was defined, based on the average of the range drawn by all subjects. Also, the midpoint of the centers of both ROIs was defined as the center of each tool (Figure 2A). The cumulative fixation time on the ROI for functional parts for each subject was extracted for each condition and familiarity. This was achieved using the analysis software, Tobii Studio. Finally, the cumulative fixation time on the functional part of the tool served as a quantitative indicator of the degree of technical reasoning.

#### Visualization of Time-Series Gaze Movement and Cumulative Fixation Position

For the eye movement data, we standardized the horizontal axis coordinates using the specified center of each tool as the origin (Figure 2A). This standardized the distance from the center to the outer edge of the functional ROI as + 100. The distance to the outer edge of the grasping ROI was standardized as −100. Data outside of both ROIs were treated as missing values.

The average gaze position of each subject was calculated for each condition and familiarity level. This took place every 250 ms from the standardized eye movement data. The average gaze position of all subjects was calculated and coordinated to visualize the movement of gaze points over time. Moreover, histograms (number of bins: 10) were created for each condition and familiarity level. This was based on the number of measurements of gaze data at standardized horizontal axis coordinates for all subjects. Finally, MATLAB R2017b was used to process these data.

### Statistical Analysis

A Mann–Whitney’s *U*-test was performed to test the degree of familiarity of the six familiar and the six unfamiliar tools.

The ROI areas for the functional part of familiar and unfamiliar tools were compared using the *t*-test (first, the *F*-test and then the Welch’s test).

A two-way analysis of variance for tools (familiar and unfamiliar) and conditions (free to see, lift, and use) was conducted on the cumulative fixation time of the ROI for the functional part. A multiple comparison test was also undertaken, using the Shaffer method.

The statistical software, R version 3.4.1, was used for these statistical processes. The significance level was set at 5%.

## Results

### Degree of Familiarity

Familiar tools had a higher degree of familiarity than unfamiliar tools (*P* < 0.01). The median value of the familiar tools was 5 or 4, the maximum was all 5, and the minimum was 4 or 5. The median value of all unfamiliar tools was 1, the maximum value was either 1 or 3, and the minimum value was all 1.

### Region of Interest for the Functional Part and Region of Interest for the Grasping Part

Figure 2B shows the ROI for the functional part, the ROI for the grasping part, and the center of the tool calculated from the average of the range drawn by the subjects.

#### Region of Interest Area of the Functional Part

There was no significant difference in the ROI area of the functional part between familiar and unfamiliar tools (*P* = 0.3575).

#### Heat Map of All Fixations and Cumulative Fixation Position

**FIGURE 3:**
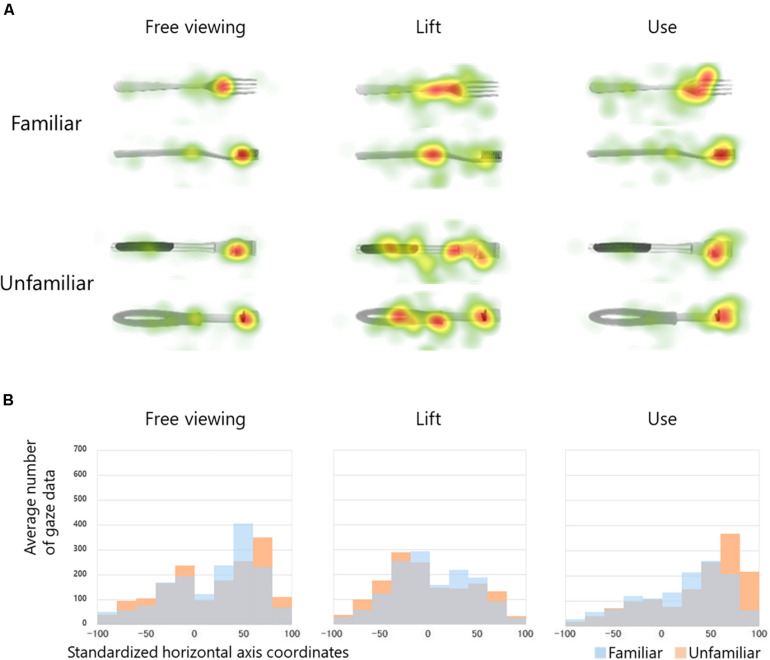
**(A)** Heat map of all fixations. Top two rows: two excerpts from familiar tools; bottom two rows: two excerpts from unfamiliar tools. Left: free viewing conditions; center: lift conditions; right: use conditions. Heat maps for each condition created based on all fixations (two familiar tools and two unfamiliar tools). Areas with a long fixation time are indicated in red. **(B)** Histogram of cumulative fixation position. Vertical axis shows the average number of gaze data in each bin, and the horizontal axis indicates the standardized horizontal coordinates.

Figure 3A shows the heat maps for each condition, based on all fixations for the two familiar and unfamiliar tools. Compared to the lift conditions, the fixations in the use and free viewing conditions were biased toward the functional parts.

Figure 3B is a histogram of the cumulative gaze position average, based on gaze data with standardized horizontal axis coordinates. The bias of the gaze in the direction of the functional part was observed under the use and free viewing conditions, though not the lift condition. Moreover, under the use conditions, the gaze was deflected to the functional part for the unfamiliar tools more so than the familiar ones.

### Cumulative Fixation Time on Functional Parts

**FIGURE 4:**
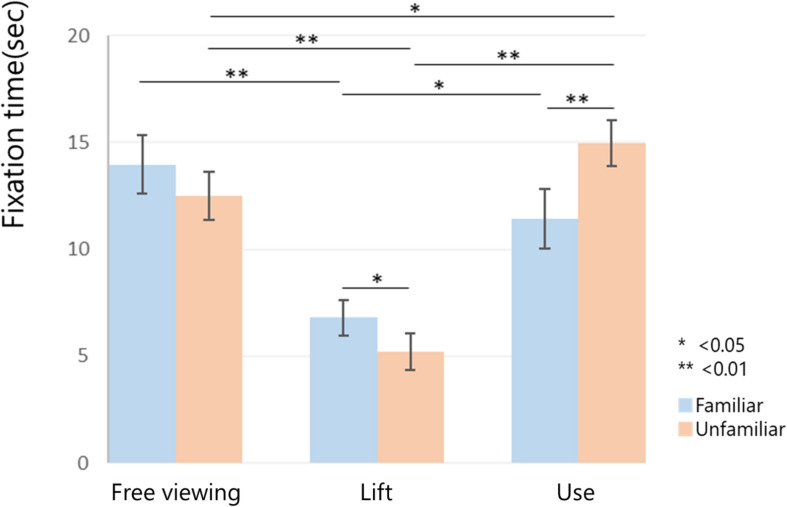
The cumulative fixation time on the functional part. The cumulative fixation time on the functional ROI for each condition and familiarity is shown. We found a main effect for conditions (*F*(2,26) = 28.2112, *P* < 0.01) and no main effect for the familiarity (*F* = (1,13) = 0.0903, *P* = 0.7686). The cumulative fixation times under the free viewing and use conditions significantly increased, compared to those under lift conditions (*P* < 0.01). Furthermore, there was an interaction between condition and familiarity (*F*(2,26) = 9.3635, *P* < 0.01). Relative to the free viewing conditions, the cumulative fixation time for unfamiliar tools significantly decreased during the lift conditions (*P* < 0.01) and significantly increased during the use conditions (*P* < 0.05). *P < 0.05; **P < 0.01.

The two-way analysis of variance showed a main effect on conditions (*F*(2,26) = 28.2112, *P* < 0.01). The cumulative fixation times under free viewing and use conditions significantly increased, compared with those under lift conditions (*P* < 0.01). There was no main effect on familiarity (*F* = (1,13) = 0.0903, *P* = 0.7686). There was an interaction between condition and familiarity (*F*(2,26) = 9.3635, *P* < 0.01). The cumulative fixation time increased for the unfamiliar tool under the use conditions (*P* < 0.01), while the cumulative fixation time increased for the familiar tool under the lift conditions (*P* < 0.05). The cumulative fixation time for the familiar tool had a significantly greater increase during the free viewing (*P* < 0.01) and use conditions (*P* < 0.05) than it did during the lift conditions. Furthermore, relative to the free viewing condition, the cumulative fixation time for unfamiliar tools significantly decreased during the lift conditions (*P* < 0.01) and significantly increased during the use conditions (*P* < 0.05) (Figure 4).

#### Visualization of Time Series Gaze Movement

**FIGURE 5:**
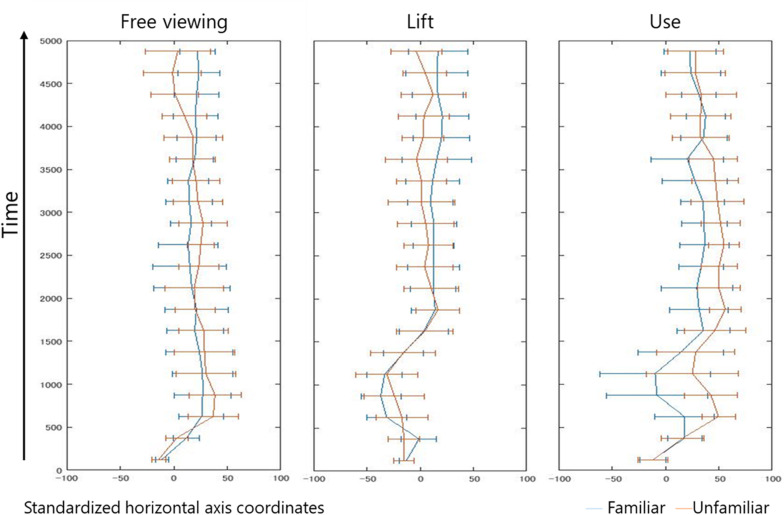
Time series change in mean gaze position. The vertical axis indicates time, and the horizontal axis indicates the standardized horizontal coordinates. The figure shows the average gaze position every 250 ms. Error bars indicate the standard deviation.

Figure 5 shows a graph of the average gaze position movement every 250 ms and a histogram using the cumulative gaze position average, based on gaze data with standardized horizontal axis coordinates. The bias of the gaze in the direction of the functional part was visualized more under the use and free viewing conditions, compared with the lift condition. Under the use conditions, the mean fixation position moved across the center and toward the grasp (only with the familiar tools) at 750–1,000 ms and 1,000–1,250 ms.

## Discussion

There was no significant difference between familiar and unfamiliar tools in ROI area – which was extracted from the mean of the functional part drawn by all subjects. This suggests that the difference in the area between tools did not affect the difference in cumulative fixation time for the ROI of the functional part. The degree of familiarity was significantly higher for familiar tools than for unfamiliar tools. This suggests that our selection of tools was reasonably familiar. In cumulative fixation time, there was a main effect on the condition, but no main effect on the familiarity of tools. A multiple comparison test showed that the use and free viewing conditions resulted in a greater increase in cumulative fixation time on the functional parts, compared with the lift conditions. This result is consistent with a previous study (Belardinelli et al., 2016), which showed that the gaze was biased toward the functional part of the tool only when the subject was asked to use it. In our results, this was not found to be the case when the subject was asked to lift the tool, regardless of familiarity, namely, this result indicates that, similar to what happened in a prior study (Myachykov et al., 2013), the intention to lift caused participants to prioritize looking at the grasping part – an action meant to infer the lifting of the tool – over its functional part – an action meant to infer tool use. Furthermore, the amount of time spent looking at functional parts increased during free viewing (without the intention to use) more so than it did during the intention to lift. This suggests that participants’ reasoning work was similar during free viewing and viewing with the intention to use the tool. However, it might be possible that participants only inspected the functional part first, namely, the search for the characteristic functional part of the tool occurred in the free viewing condition, but not in the lift condition. However, in the use condition (i.e., the last one), we observed the greater gaze bias toward the functional part of the unfamiliar tool compared with other conditions; this suggests that the necessity of technical reasoning increases with the intention to use the tool. To make it clearer that technical reasoning is automatically activated in the free viewing condition owing to an automatic inference toward use, future researchers could present two conditions (i.e., the lift and free viewing conditions) at random. Future studies should endeavor to explore if technical reasoning is indeed activated in the free viewing condition owing to an automatic inference toward tool use, researchers could present the lift and the free viewing conditions at random.

We also found an interaction between condition and familiarity. Under the use conditions, participants spent more time looking at the functional parts of unfamiliar tools than those of familiar tools. This suggests that, depending on the intention of use, participants are more deliberate about observing the mechanical structure of the functional part and analogizing its use (i.e., technical reasoning). Furthermore, this increased deliberation is more pronounced for unfamiliar tools than familiar ones. On the other hand, under the lift conditions, participants spent more time looking at the functional parts of familiar tools than unfamiliar ones, namely, although the intention to lift led subjects to prioritize looking at the grasping part of the tool (an action meant to infer the lifting of the tool) over its functional part (an action meant to infer tool use), we observed that, under the lift conditions for familiar tools, participants had greater gaze bias toward the functional part; still, we highlight that this may owe to a higher-order affordance effect based on functional knowledge, as was remarked in a past study (Van Der Linden et al., 2015). This is corroborated by the fact that, even with familiar tools, the time spent looking at the functional parts was longer during the use and free viewing conditions than the lift conditions. Thus, there may have indeed been less gaze bias toward the functional parts upon intention to lift, that is, participants prioritized looking at the grasping part of the tool – an action meant to infer the lifting of the tool – over its functional part – an action meant to infer tool use. This may also be supported by the finding that, for unfamiliar tools, the time spent looking at the functional parts was longer during the use and free viewing conditions than the lift conditions.

Importantly, there was an apparent gaze bias toward the functional parts even during free viewing, and it is likely that participants were automatically doing reasoning work for use – even if they did not intend to act on it. The gaze bias toward the functional part during free viewing may reflect a process of preparation for potential use that is automatically initiated during the observation of the tool (Lewis, 2006). For familiar daily tools, this is an automatic semantic reasoning based on functional knowledge (evoked from the shape of the functional part), as shown in a past study (Van Der Linden et al., 2015); for unfamiliar tools, this is an automatic technical reasoning afforded from the mechanical characteristics of the shape of the functional part – a reasoning meant to analogize the usage method.

There was no difference between the free viewing and use conditions for the familiar tools; nonetheless, the fixation time on the functional part was longer during the use conditions than the free viewing conditions for the unfamiliar tools. These results on familiar tools may indicate that knowledge on tool function leads to automatic preparation for use during free observation – in which people are not required to use the tool. Despite this lack of intent for use, the preparation is almost equal to that observed in the condition where people had the intent to use the tool. However, in the time-series gaze position shift, there was a gaze shift in the direction of the grasping part that took place approximately 1,000 ms after the presentation. This gaze shift may reflect the subjects’ confirmation of the actual part that they will need to grasp to use the tool. This may support findings that the gaze shifts occur first toward the actual grasping point, in accordance with the action intention (Brouwer et al., 2009; Belardinelli et al., 2015). Along with that, the results may indicate that free observation of the familiar tool involves automatic reasoning work, but does not evoke an image of the actual movement.

Interestingly, these results above seem to contradict prior studies; two research found that participants gazed more at the grasping part when they could freely observe a familiar tool and an object with a consistent combination (Federico and Brandimonte, 2019, 2020). However, in one of these studies, when the combination was consistent and presented at a spatially distant location, participants gazed more at the functional part of the tool (Federico and Brandimonte, 2019), namely, if the tool and the combined object are not in a spatial position where they can be manipulated, people may not automatically imagine the action of using the tool. Thus, these specific results seem consistent with our results. Furthermore, in our study, we did not present another object in combination with the tool; instead, we presented only the tool. This experimental condition corresponds methodologically to the free viewing condition of Federico and Brandimonte (2020), in which the object and the tool are inconsistently combined. Moreover, the short-term recognition task of tools (or objects) present in Federico and Brandimonte (2020)’s study corresponds methodologically to the use condition in the current study, since it involves the process of recalling how to use the tools (or objects). The results of these conditions in Federico and Brandimonte (2020)’s study are consistent with the results of the free viewing and use conditions in our study – an increase in gaze on the functional parts of the tools. Therefore, our results are consistent with those of Federico and Brandimonte (2020)’s study.

The results for unfamiliar tools reveal that, although technical reasoning is still present in free observation, it is present to a lesser degree than when the subject intends to use the tools. When we are asked to use a tool, there is a need to actively extract its mechanical properties to guess how it will be used. Such a need does not arise during free observation. This difference may represent the difference between intentional and automatic technical reasoning. Thus, in a tool observation task with different degrees of familiarity, discerning the degree of technical reasoning may depend on the distinction between viewing with intention to use and free viewing.

In the future, it is important to compare the characteristics of gaze search for familiar and unfamiliar tools in patients with tool use disorder; specifically, we suggest research exploring the pathological mechanisms of tool use disorder. Methodologically, this can be done by conducting comparisons at the time of request for use and during free viewing; if the gaze bias toward the functional area does not change between the use and free viewing conditions, the lack of technical reasoning may be one of the causes of tool use disorder. On the other hand, if the gaze bias is increased, it may be inferred that the patient is trying to make technical reasoning, although with some struggle. Moreover, patients with tool use disorder often show left hemisphere brain damage, so there is a tendency to motor paralysis of the right hand (i.e., usually the dominant hand) in this population; this leads to a propensity to perform tool use with the left hand (i.e., non-dominant hand). In our experiment, to reduce differences in motor effort, tool orientation was standardized so that right-handed participants performed tool use with the left hand; it was likely that participants had greater experience with using their right hands to manipulate familiar tools, which would evoke lesser motor effort compared with using the left hand. Thus, we believe that our present results – found based on methods that ensured participants would perform tool use with the non-dominant hand – may be useful for future comparisons with studies that conduct similar experimentations in patients with tool use disorder.

In this study, however, the actual use of tools was not the ultimate intention. Indeed, the aim was for participants to pantomime as if they were using the tools. Previous studies show that there were differences in fixation point characteristics between actual tool use and pantomime (Kobayakawa et al., 2007), and patient studies revealed a symptom of dissociation between actual tool use and pantomime (Motomura and Yamadori, 1994; Fukutake, 2003). Therefore, it should be noted that the results obtained by the application of this task require careful consideration. Additionally, to explore the possibility of bias toward gazing at the functional part of the tool first and overall attentional bias toward the right side of the screen, future studies are warranted to randomize and examine condition order and tool orientation.

## Conclusion

The cumulative fixation time on the functional part of the tool during free viewing and intention to use was significantly higher than that during intention to lift. Thus, during intention to lift, participants prioritized looking at the grasping part of the tool – an action meant to infer the lifting of the tool – over its functional part – an action that denotes reasoning work to use the tool; during free viewing, the reasoning work for using the tool was automatically performed. Cumulative fixation time for unfamiliar tools showed a significantly greater increase during free viewing compared with during intention to lift; this increase was also significantly greater during intention to use compared with during free viewing. Thus, the technical reasoning taking place during free viewing is not as intense as that during intent to use. It was suggested that the difference in technical reasoning between free viewing and intention to use may be reflective of the difference between automatic and intentional technical reasoning.

## Data Availability Statement

The raw data supporting the conclusions of this article will be made available by the authors, without undue reservation.

## Ethics Statement

The studies involving human participants were reviewed and approved by the Research Ethics Committee of Kio University. The patients/participants provided their written informed consent to participate in this study.

## Author Contributions

YTam designed the study, collected and analyzed the data, and wrote the manuscript. YTak, YM, MT, and SM provided experimental equipment and helped with data analyses. SN and SM supervised the study. All authors read and approved the manuscript.

## Conflict of Interest

The authors declare that the research was conducted in the absence of any commercial or financial relationships that could be construed as a potential conflict of interest.
